# The Impact of Childhood Trauma on Neuropsychological Disorders and Its Intergenerational Transmission Mechanisms

**DOI:** 10.3390/bs16071104

**Published:** 2026-07-03

**Authors:** Xuyan Cheng, Ping Liu, Qing Zhang

**Affiliations:** School of Mental Health and Psychological Sciences, Anhui Medical University, Hefei 230032, China; 2213240089@stu.ahmu.edu.cn (X.C.); 2445011147@stu.ahmu.edu.cn (P.L.)

**Keywords:** childhood trauma, intergenerational transmission, neuropsychological disorders

## Abstract

Childhood trauma is adversely linked to a spectrum of physical and neurobehavioral disorders, further facilitating the intergenerational transmission of familial trauma. This review systematically elaborates on the profound impacts of childhood trauma on both survivors and their descendants. It provides an in-depth analysis of the complex mechanisms underlying this intergenerational transmission, and innovatively proposes an Environmental-Psychological-Physiological-Molecular (EPPM) multilevel cross-generational interaction model. This model encompasses behavioral transmission of negative parenting, neural encoding of traumatic stress, intergenerational neurophysiological basis, biological permeation of the intrauterine environment, and mechanisms of epigenetic remodeling. This provides a scientific basis for deepening our understanding of the long-term consequences of trauma, promoting the development of early intervention strategies from an interdisciplinary perspective, and breaking the intergenerational cycle of trauma.

## 1. The Prevalence and Lifelong Impact of Childhood Trauma

Childhood trauma (CT) refers to stressful or traumatic events experienced during childhood (Gilgoff et al., 2020), which are generally classified into two distinct categories: threat-related trauma and deprivation-related trauma. Threats include emotional abuse (EA), physical abuse (PA), and sexual abuse (SA), while deprivations include emotional neglect (EN) and physical neglect (PN). EA includes verbal abuse and manipulative behavior, such as placing a child in situations that induce shame, guilt, or fear to satisfy the abuser’s emotional needs, or persuading a child to act against their will. EN refers to the failure to meet a child’s basic emotional needs. PN refers to the failure to provide a child’s basic necessities, such as adequate food, appropriate clothing, a safe living environment, proper supervision, and necessary medical and dental care (Teicher & Samson, 2016). In China, the phenomenon of child neglect is particularly severe, with a prevalence rate of 78.6% for PN and 60% for EN. These figures are significantly elevated relative to the corresponding rates in Europe. In contrast, the prevalence of child abuse in China is relatively low, with PA occurring at a rate of approximately 36.6% and SA at approximately 18.6% (Lu et al., 2020).

Childhood trauma is linked to negative effects on the physical and mental health of those who experience it, and is accompanied by an elevated risk of developing various physical and mental health conditions. Retrospective and prospective studies indicate that experiences of childhood trauma are associated with depressive and anxiety disorders in children (Copeland et al., 2007; M. Li et al., 2016; Raposo et al., 2014), attention deficit hyperactivity disorder (ADHD) (Rodriguez et al., 2025), post-traumatic stress disorder (PTSD) (Copeland et al., 2007), and Body Dysmorphic Disorder (Monzani et al., 2024). In addition, childhood trauma has also been linked to the development of personality disorders in adulthood (Raposo et al., 2014). Individuals with a history of childhood abuse or neglect exhibit an elevated risk of chronic pain (e.g., migraines) (Burke et al., 2017; Bussières et al., 2023; Nicolson et al., 2023; Tietjen et al., 2010), obesity (Mundi et al., 2021), asthma (Scott et al., 2008), cardiovascular stress responses (Voellmin et al., 2015), coronary heart disease (Deschênes et al., 2021), acquired brain injury (Guinn et al., 2019), endocrine disorders (Voellmin et al., 2015), inflammation and physiological dysregulation (Cornman et al., 2023), and cancer in adulthood (Ports et al., 2019). Furthermore, traumatic childhood experiences are associated with the occurrence of self-harming behaviors, such as alcohol abuse, smoking, risky HIV behaviors (J. A. Campbell et al., 2016), substance abuse, suicide attempts, and sexually transmitted infections (Wade et al., 2016). In summary, adverse childhood experiences (ACEs) have lasting detrimental effects that persist throughout a person’s life, causing immeasurable and irreversible harm (Hughes et al., 2017). Childhood trauma is not only associated with negative effects on an individual’s physical and mental health but is also linked to issues such as increased crime and the excessive consumption of social resources. Reports indicate that millions of adults in Europe and North America live with the lingering effects of childhood trauma. A 10% reduction in the prevalence of childhood trauma could result in annual savings of $105 billion (Bellis et al., 2019). In summary, understanding and intervening in childhood trauma and its mechanisms are critical to alleviating its far-reaching detrimental consequences for individuals, families, and society.

## 2. Intergenerational Links Between Parental Childhood Trauma and Children’s Neuropsychological Disorders

**Figure 1:**
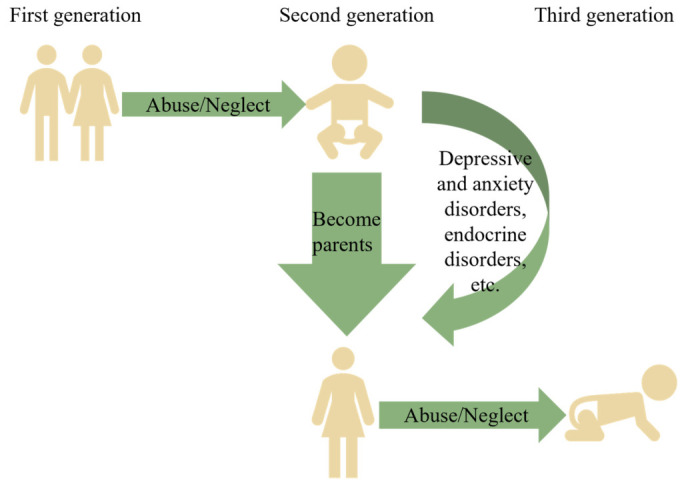
Mechanisms of intergenerational transmission of childhood trauma.

The effects of childhood trauma are not confined to the children who experience traumatic events. They also extend to the next generation, affecting a larger population (as shown in Figure 1). This is known as the intergenerational effect or the generational cascade effect (Q. Zhang et al., 2017). Research indicates that parental childhood trauma is associated with high levels of internalizing problems (Giallo et al., 2020; Roberts et al., 2015), externalizing problems (Liu et al., 2019; Miranda et al., 2013; W. Li et al., 2020), and cognitive impairments in offspring (Lyons-Ruth et al., 2023). Children raised by parents with a history of childhood trauma are more likely to develop ADHD (Moon et al., 2021), autism spectrum disorder (Moog et al., 2023), and mental or neurological conditions such as depression and anxiety disorders (Kang et al., 2021). In addition, these children may face an elevated risk of developing conditions such as obesity (Leonard et al., 2017), allergies (Tomfohr-Madsen et al., 2016), and asthma (Tomfohr-Madsen et al., 2016). Several studies suggest that maternal contributions to child development outweigh paternal ones (Oshio & Umeda, 2016). For example, compared with paternal ACEs, maternal ACEs show a stronger association with child problem behaviors (Schickedanz et al., 2018). However, other studies have found that maternal childhood abuse is more strongly associated with problem behaviors in female offspring, while paternal childhood abuse is more closely related to such behaviors in male offspring, indicating a same-sex transmission pattern of childhood abuse to offspring problem behaviors (Oshio & Umeda, 2016). Emerging evidence demonstrates that paternal ACEs also confer significant intergenerational risk. A recent meta-analysis of 19 studies found that paternal ACEs were positively associated with child behavioral problems (r = 0.15) and child ACEs (r = 0.26), indicating a small-to-moderate transmission effect (Deneault et al., 2025). Moreover, paternal ACEs have been linked to lower parenting satisfaction and higher depressive symptoms, which may indirectly affect child outcomes (Grafft et al., 2025). These findings underscore that paternal childhood adversity independently contributes to offspring neuropsychological vulnerability, warranting greater inclusion of fathers in intergenerational research. Therefore, a comparative examination of the mechanisms underlying childhood trauma experiences in both parents may be valuable.

Maternal childhood trauma is associated with infant brain development. Studies indicate that compared to mothers without such experiences, those with a history of childhood trauma tend to have newborns with reduced brain volumes (Moog et al., 2018). This association has been repeatedly observed across infant samples. Lyons-Ruth et al. (2023) observed that more severe childhood abuse experienced by mothers was linked to smaller gray matter volume in their infants, with this association persisting throughout early development. Reduced gray matter volume may be associated with certain mental illnesses (X. Wang et al., 2018), reflecting neuronal damage or a reduction in synapses in the infant. In addition to the macroscopic indicator of brain gray matter volume, studies have also identified specific associations between maternal childhood trauma and emotion-related brain regions (the amygdala and hippocampus). Maternal ACEs are associated with smaller amygdala volume (Demers et al., 2022; Lyons-Ruth et al., 2023). Elevated maternal stress levels during pregnancy are associated with reduced hippocampal volume in newborns and a decelerated hippocampal growth rate throughout the initial six months postpartum (Nolvi et al., 2023). The hippocampus, characterized by a high density of glucocorticoid receptors, may be particularly vulnerable to the “toxic effects” of glucocorticoids, which can contribute to the loss of neurons and synapses (Humphreys et al., 2019). Consequently, reduced hippocampal volume is associated with mental disorders such as PTSD and major depressive disorder (Barch et al., 2019; Smith, 2005). Functionally, the effective operation of the amygdala–ventromedial prefrontal cortex circuit helps suppress negative emotions and facilitates sound reward-related decision-making (Hiser & Koenigs, 2018). In contrast, newborns of mothers who experienced childhood abuse or neglect show increased connectivity between the amygdala and frontal regions (Hendrix et al., 2021). These alterations in amygdala function may represent an adaptive developmental response to early adversity (van den Heuvel et al., 2023).

Maternal ACEs are also associated with hormonal stress responses in the next generation. For example, maternal childhood trauma is linked to elevated cortisol levels in children’s hair (Slopen et al., 2018), and to abnormal hypothalamic–pituitary–adrenal (HPA) axis activity in the offspring (Molenaar et al., 2019). In male offspring, maternal ACEs may correlate with adrenal hypoplasia (Duffy et al., 2023).

In summary, the consequences of childhood trauma are influenced by multiple factors, including trauma type, individual coping, timing and duration of exposure, and family and social environment. Furthermore, the number of abuse types mothers experience is directly related to the health risks for their offspring, demonstrating a clear dose–response relationship (Tomfohr-Madsen et al., 2016).

## 3. Intergenerational Transmission Mechanisms of Childhood Trauma on Neuropsychological Disorders

Studies indicate that maternal childhood trauma significantly increases the risk of developmental delays in offspring via various biological and psychosocial pathways (Ishikawa et al., 2022). However, the mechanisms underlying intergenerational transmission remain poorly understood.

This paper systematically summarizes the key mechanisms and proposes an Environmental–Psychological–Physiological–Molecular (EPPM) multilevel cross-generational interaction model.

**Figure 2:**
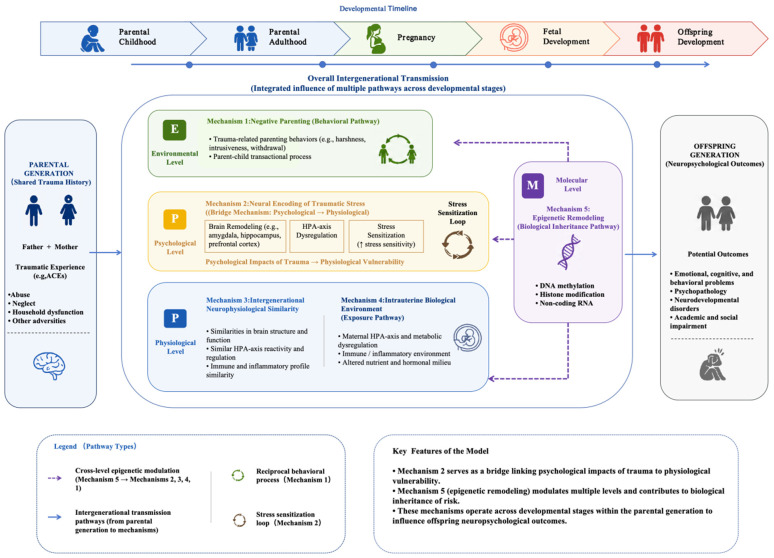
The Environmental–Psychological–Physiological–Molecular (EPPM) developmental framework for the intergenerational transmission of childhood trauma. Note. The EPPM framework conceptualizes intergenerational trauma transmission as a multilevel developmental process spanning environmental, psychological, physiological, and molecular domains. The pathways shown represent proposed mechanisms derived from current evidence and are intended to guide future research and intervention development.

The model integrates and extends the prenatal biological framework of Buss et al. (2017) and the biopsychosocial mediators reviewed by Ishikawa et al. (2022) by placing behavioral, neurophysiological, intrauterine, inflammatory, and epigenetic mechanisms within a dynamic developmental framework. As illustrated in Figure 2, the EPPM model proposes a temporal sequence in which parental childhood trauma may first become biologically embedded through neural encoding and stress-related neurophysiological changes. These changes may then influence parenting behaviors, the intrauterine environment, and offspring neurodevelopment across developmental stages. The model also emphasizes that these mechanisms are interconnected rather than isolated. In particular, parent–child interactions may involve reciprocal processes, whereby offspring’s emotional or behavioral difficulties increase parenting stress and further reinforce maladaptive parenting behaviors. Genetic and epigenetic mechanisms are conceptualized as cross-level modulators that shape individual differences in vulnerability and intergenerational transmission.

To enhance the transparency of this narrative review, the literature search and selection process adhered to the following principles:

1. Databases consulted: PubMed, PsycINFO, Web of Science, and Google Scholar were systematically searched.
2. Search period: The search covered publications from January 2000 to October 2024, with priority given to recent high-quality evidence.
3. Keywords and themes: The content in the Table 1 indicates the keywords for exploring the mechanisms of childhood trauma.
4. Study selection rationale: Given the narrative nature of this review, we prioritized peer-reviewed original research, systematic reviews, and meta-analyses that provided mechanistic insights into intergenerational transmission. Non-English articles and studies lacking clear empirical or theoretical contributions to transmission mechanisms were excluded.

### 3.1. Mechanism 1: Transmission of Negative Parenting Behaviors

Traumatic experiences may be transmitted across generations due to the close association between maternal childhood trauma and her negative parenting styles toward her offspring (Lotto et al., 2023). For instance, while caring for her child, a mother may be triggered by past traumatic experiences, leading her to unconsciously inflict abuse on the child (Amos et al., 2011). Therefore, compared to parents without a history of childhood trauma, those with such a history are more likely to abuse their own children (Hughes et al., 2017). Mothers who have experienced violence or PA are more likely to use corporal punishment than mothers without such experiences (Chung et al., 2009). Women with a history of SA tend to engage in physical abuse or corporal punishment when parenting their children (Schuetze & Eiden, 2005). Due to cumulative effects, the frequency and variety of parental childhood maltreatment are positively associated with the risk of child maltreatment in the subsequent generation (Greene et al., 2020). Mothers who have experienced EA tend to interpret their children’s facial expressions as expressions of negative emotions (Pétrin et al., 2024). Conversely, mothers with a history of SA tend to rely prematurely on their children for emotional support (Schuetze & Eiden, 2005). Beyond maternal interpretation, parental sensory processing sensitivity has also been linked to children’s atypical attentional patterns to emotional faces (Christou et al., 2024). These factors may be closely linked to their abusive behaviors.

Maternal history of childhood abuse is associated with abusive behavior toward offspring, which in turn affects their developmental outcomes. For example, research indicates that maternal abuse of offspring mediates the relationship between maternal history of childhood abuse and children’s behavioral adjustment problems and competence (Kızıltepe & Yılmaz Irmak, 2024). Maternal childhood trauma is positively associated with parenting stress, which exacerbates child abuse and neglect and subsequently undermines children’s early executive function (Zha et al., 2024). Fathers with ACEs also display less optimal parenting: higher paternal ACE scores are associated with lower fathering satisfaction, an effect partially mediated by paternal depressive symptoms (Grafft et al., 2025). At the same time, children’s emotional and behavioral difficulties may further increase parenting stress and reinforce maladaptive parent–child interactions, suggesting a reciprocal feedback loop rather than a purely unidirectional pathway (S. Wang & Gai, 2024; Remondi et al., 2024). Most evidence supporting this pathway comes from observational human studies. Cross-sectional and longitudinal findings consistently suggest that parental childhood trauma is associated with maladaptive parenting and offspring difficulties, but residual confounding factors, including parental psychopathology and shared environmental risks, cannot be fully excluded.

### 3.2. Mechanism 2: Neural Encoding of Traumatic Stress

Evidence for the neural encoding of traumatic stress comes from human neuroimaging studies, longitudinal developmental cohorts, and experimental stress models. Childhood trauma is significantly associated with neurophysiological development. This association can be summarized in two aspects: first, its relation to alterations in brain structure and function; and second, how it, as a stressor, causes functional changes in the HPA axis.

#### 3.2.1. Changes in Brain Structure and Function

Childhood abuse may be linked to specific alterations in brain regions and fiber tracts that process and transmit abuse-related negative sensations. Certain brain structures that are particularly susceptible to childhood abuse are often accompanied by the following characteristics: (1) prolonged postnatal development; (2) high density of glucocorticoid receptors; (3) some degree of postnatal neurogenesis (Teicher et al., 2003; Teicher & Samson, 2016). This view is supported by empirical evidence. For instance, childhood SA is associated with significant cortical thinning in the genital representation area of the primary somatosensory cortex. In comparison, EA is associated with cortical thinning in regions related to self-awareness and self-evaluation (Heim et al., 2013). Early trauma may also be associated with alterations in cortical regions involved in emotional processing and stress responses, thereby altering the normal trajectory of brain development (Teicher et al., 2016).

Some studies report that abuse or neglect is associated with reduced amygdala volume (Hanson et al., 2015), while others report that individuals who have experienced parental neglect exhibit larger amygdala volumes (Mehta et al., 2009; Tottenham et al., 2010). These contradictory findings likely arise from multiple sources, including the type of adversity examined (e.g., threat-based abuse vs deprivation-based institutional care), sample characteristics (e.g., family-reared children vs. previously institutionalized children and amygdala segmentation approaches (e.g., hand-tracing vs. automated segmentation). In addition, the timing of stress exposure may play a critical role. The amygdala develops rapidly during the first year of life, with the highest rate of structural and functional changes occurring before puberty (Fareri & Tottenham, 2016). Pechtel et al. (2014) found that adversity experienced by children aged 10–11 is correlated with increased right amygdala volume in adulthood, but not with the left amygdala volume. This lateralization may be explained by the right amygdala’s role in processing negative emotions and left amygdala processes positive emotions.

In contrast to amygdala findings, reports of reduced hippocampal volume are consistent among adolescents and adults who have experienced trauma (Hanson et al., 2015). However, findings are inconsistent among children who experienced early abuse or neglect, suggesting a latent period between adversity exposure and negative outcomes (Teicher & Samson, 2016).

Furthermore, multiple studies have reported significant gender differences. For example, male hippocampal volume appears to be more susceptible to stress effects than female hippocampal volume. In addition, the corpus callosum appears most vulnerable to neglect in males and to SA in females (Everaerd et al., 2012; McEwen, 2010; Samplin et al., 2013). In females, abnormalities in resting-state functional connectivity between the anterior cingulate cortex and the hippocampus or amygdala may be more pronounced, a phenomenon that may explain why women have an elevated risk of anxiety and depression (Herringa et al., 2013). Most evidence regarding trauma-related brain alterations is derived from observational neuroimaging studies and therefore supports neural associations rather than definitive causal mechanisms. Longitudinal neuroimaging studies provide stronger evidence for developmental trajectories, but they cannot completely eliminate the influence of genetic and environmental confounding. Emerging longitudinal evidence suggests that trauma-related neural alterations may not only reflect the consequences of early adversity but may also increase sensitivity to later stressors, thereby contributing to a dynamic developmental adaptation process rather than a single linear pathway.

#### 3.2.2. Hypothalamic–Pituitary–Adrenal Axis Function

Under normal physiological conditions, the hypothalamic–pituitary–adrenal (HPA) axis regulates the body’s stress response. Cortisol, the end product of the HPA axis, plays a central role in responding to physical and psychological stressors. Elevated circulating cortisol levels suppress the release of corticotropin-releasing hormone (CRH) from the hypothalamus and adrenocorticotropic hormone (ACTH) from the pituitary gland, via binding to mineralocorticoid and glucocorticoid receptors, thereby exerting negative feedback and reducing cortisol secretion (Levine et al., 2007).

Early childhood trauma, as a form of chronic stress, initially hyperactivates the HPA axis (Kaess et al., 2018). This chronic activation of the HPA axis results in the release of glucocorticoids throughout the brain and body. However, early-life stress can disrupt the balance of the HPA axis, which may increase susceptibility to mental disorders (Juruena et al., 2021). A dysregulated HPA axis typically exhibits either persistent hyperreactivity or hyporeactivity, which is associated with a reduced cortisol arousal response, a flattened diurnal cortisol slope, and elevated nocturnal cortisol levels (Rovnaghi et al., 2021). Brain regions with high densities of glucocorticoid receptors, such as the prefrontal cortex and hippocampus, are particularly sensitive to glucocorticoid exposure. Their vulnerability to such exposure may impair neural plasticity in these areas (K. A. Campbell, 2022). Evidence suggests that the cortisol response to stress is related to the acute intensity and severity of stress, and chronic early-life stress may sensitize the HPA axis, leading to neuroendocrine function (Nandam et al., 2019). HPA axis dysfunction is a hallmark of major depressive disorder (Zajkowska et al., 2022). Importantly, compared with adult psychopathology, HPA axis dysfunction is more closely linked to childhood trauma (Carvalho Fernando et al., 2012). Thus, individuals with a history of childhood trauma may develop altered HPA-axis functioning and increased stress sensitivity, which may heighten vulnerability to subsequent stress exposure and contribute to further neurobiological adaptation across development. This dynamic process is consistent with the stress sensitization and latent vulnerability frameworks. Fathers exhibit caregiving-related neural plasticity: reduced orbitofrontal gray matter correlates with greater intrusiveness, and decreased precuneus volume predicts stronger neural responses to own infant cues (Kim et al., 2014; Paternina-Die et al., 2020). Human evidence linking childhood trauma to HPA-axis dysfunction is largely observational, although prospective studies support stress sensitization across development. Experimental animal models further strengthen mechanistic interpretation by demonstrating that early-life stress can directly alter glucocorticoid signaling and stress responsivity.

### 3.3. Mechanism Three: Intergenerational Neurophysiological Foundations

Evidence for intergenerational neurophysiological foundations is derived primarily from observational neuroimaging and psychophysiological studies. The similarity in neural structure and function between parent and child, as well as the intergenerational association of HPA axis function between mother and fetus, is closely associated with susceptibility to psychiatric disorders. This may represent a mechanism underlying intergenerational transmission at the neurophysiological level.

#### 3.3.1. Neurophysiological Similarity Between Parents and Children

Recent studies on social anxiety disorder (Bas-Hoogendam et al., 2018), depression (Foland-Ross et al., 2016; Minami et al., 2022), dyslexia (Vandermosten et al., 2020), and ADHD (Poissant et al., 2014) indicate that neural similarity may be stronger in parent–child pairs at risk for psychopathology. As Foland-Ross and colleagues found, cortical thickness in the left fusiform gyrus, inferior temporal gyrus, and lateral occipital cortex in mothers with a history of depression directly predicted cortical thickness in high-risk daughters. In contrast, no such association was observed between non-depressed mothers and their low-risk daughters (Foland-Ross et al., 2016). Taken together, parent–child neural similarity varies as a function of risk factors.

Based on attachment theory and social learning theory, researchers propose that the human brain develops through a process of “biobehavioral synchronization.” In this process, parents and children continuously exchange behavioral, hormonal, and physiological signals during social interactions, and neural similarity may undergo adaptive changes. Intergenerational neuroimaging studies indicate that neural similarity between mothers and children exceeds that between randomly matched adults and children (Ahtam et al., 2020; Fehlbaum et al., 2022). Moreover, within parent–child pairs, similarity in cortical sulcal patterns and cortical surface area exceeds that in cortical thickness and gray matter volume. This likely reflects that sulcal morphology and surface area develop earlier and are more strongly genetically influenced, whereas measures such as gray matter volume and cortical thickness exhibit a more protracted developmental time course. Therefore, different brain structural and functional phenotypic measures may differ in their degree of environmental influence.

Furthermore, demographic indicators such as age, gender, and educational attainment may also be associated with the intergenerational transmission of brain structure or function. Studies have shown that as infants age, mother-infant similarity in resting-state functional connectivity increases continuously, and this similarity is greater among mothers with lower educational attainment and their children (Kim et al., 2021). Most notably, multiple neuroimaging studies have reported gender-specific patterns of intergenerational transmission. Examples include gray matter volume in limbic-cortical circuits (amygdala, hippocampus, and prefrontal cortex) associated with emotional regulation and depression/anxiety (Yamagata et al., 2016). They also cover sulcal patterns across the entire cerebral cortex (Ahtam et al., 2020), as well as gray matter structures of the default mode network and central executive network (Minami et al., 2022). Significant neural similarities were observed exclusively between mothers with a history of depression and their healthy daughters, but not in mother–son, father–daughter, and father–son pairs. Thus, mother-daughter neural similarity may serve as the neural basis for the intergenerational transmission of mental disorders. Fathers show no static neural similarity with their children, but greater direct caregiving time predicts stronger amygdala–STS connectivity, suggesting experience-dependent neural tuning (Abraham et al., 2014; Yamagata et al., 2016).

#### 3.3.2. Maternal HPA Axis Dysfunction and Neurophysiological Development in Offspring

Childhood trauma may influence HPA axis activity in pregnant women, which can give rise to imbalanced maternal cortisol levels, and these in turn may shape offspring stress responsivity (Epstein et al., 2020). CRH and glucocorticoids play key roles in regulating fetal neurodevelopment. While CRH exhibits neuroprotective effects, sustained elevated levels of stress hormones are associated with various changes in fetal brain structure. These changes include reduced cortical volume, decreased neuronal density in the limbic system, as well as abnormalities in neural circuits, synaptic plasticity, neurotransmission, and G protein-coupled receptor (GPCR) signaling (Kassotaki et al., 2021). Furthermore, these hormonal changes may also be detrimental to the normal development of the cardiovascular system, kidneys, brain, and immune system.

The association between maternal HPA axis dysfunction and neurophysiological development in offspring may also exhibit sex differences. For example, mothers who experienced adversity before puberty and exhibited lower cortisol reactivity during infant separation tended to have male offspring with reduced cortisol levels in response to stressors, whereas their female offspring exhibited elevated cortisol levels (Duffy et al., 2024). Furthermore, elevated maternal cortisol levels during pregnancy are associated with stronger connectivity between the daughter’s amygdala and regions subserving sensory processing and integration, together with the default mode network. By contrast, such connectivity between the amygdala and these brain regions is relatively weaker in boys. Stronger neonatal amygdala connectivity mediates the relationship between elevated maternal cortisol levels during pregnancy and increased internalizing symptoms in girls. However, this phenomenon was not observed in boys, revealing gender-specific neurophysiological mechanisms underlying internalizing mental disorders (Graham et al., 2019). Although these findings indicate intergenerational neural similarity, they cannot determine whether such similarity reflects genetic inheritance, shared environments, prenatal biological influences, or reciprocal parent–child interactions. Accordingly, the present model conceptualizes this mechanism as a predominantly unidirectional pathway, while acknowledging that ongoing parent–child interactions may contribute to experience-dependent neural tuning.

### 3.4. Mechanism 4: Biological Permeation of the Intrauterine Environment

Experiences of childhood trauma are linked to inflammation in adulthood (Danese et al., 2007). Pregnancy is a unique period in a woman’s life during which physiological functions undergo changes. Women who have experienced childhood trauma may carry these changes into the pregnancy process (Buss et al., 2017). Evidence for this pathway comes from both human prenatal cohort studies and experimental animal models. Human studies mainly indicate that maternal inflammatory and endocrine alterations during pregnancy are associated with offspring neurodevelopmental outcomes. Because these studies measure maternal biological states before birth and assess offspring outcomes later, they provide stronger temporal evidence than cross-sectional designs. However, they remain observational and cannot fully establish causality. Chronic exposure to excessive stress is linked to disruption of the maternal endocrine system and activation of inflammatory stress mediators, which may in turn disturb the normal intrauterine environment and alter the developmental trajectory of the fetal brain (Andescavage et al., 2017). Maternal immune activation (MIA) refers to immune system activation in the mother during pregnancy, triggered by infection, autoimmune reactions, or external immune stimuli. MIA can induce the release of pro-inflammatory cytokines (such as IL-6, TNF-α, and IL-17), which affect fetal neurodevelopment and may increase the risk of neurodevelopmental disorders in offspring (Urakubo et al., 2001; Woods et al., 2023). Rat models reveal that exposure to MIA is associated with structural changes in the offspring’s brain. Specifically, the volumes of the hippocampus, amygdala, striatum, and nucleus accumbens are reduced, whereas those of the thalamus, ventral midbrain, brainstem, and major white matter tracts are increased (Crum et al., 2017). Furthermore, MIA impairs the dopaminergic system and recapitulates behavioral abnormalities associated with psychiatric disorders in offspring (Luchicchi et al., 2016). Rhesus monkey studies demonstrate altered cognitive trajectories following MIA exposure (Vlasova et al., 2021). In contrast to human observational studies, maternal immune activation models in rodents and nonhuman primates allow direct experimental manipulation of inflammatory pathways. These experimental paradigms therefore provide stronger mechanistic evidence that prenatal immune activation can alter offspring brain development and behavior. Nevertheless, caution is warranted when extrapolating these mechanistic animal findings to human neurodevelopment because substantial cross-species differences exist in gestational timing, placental structure, immune regulation, and postnatal brain maturation. In humans, increased IL-6 concentrations in the prenatal environment correlate with enhanced glutamatergic synaptic function, affecting neurophysiological function (Mirabella et al., 2021). Studies have found that elevated maternal IL-6 concentrations during pregnancy are associated with larger volumes of the right amygdala in newborns. In addition, they are linked to stronger connectivity between the bilateral amygdala and brain regions such as the fusiform gyrus, somatosensory cortex, thalamus, anterior insula, caudate nucleus, and parahippocampal gyrus (Graham et al., 2018). Pro-inflammatory cytokines may cross the placental barrier and the blood–brain barrier, where they can activate microglia, trigger neuroinflammation, induce oxidative stress, and may contribute to neurodevelopmental disorders in offspring (Zawadzka et al., 2021). Concurrently, maternal markers, such as maternal immune cells, can cross the placenta via “vertical transfer,” influencing fetal brain development and postnatal cognitive behavior (Schepanski et al., 2018). Overall, evidence supporting the intrauterine biological pathway differs in methodological strength. Human prenatal studies mainly support associations and temporal relationships, whereas experimental animal models provide stronger evidence for causal biological mechanisms linking maternal immune activation to offspring neurodevelopment.

### 3.5. Mechanism 5: Epigenetic Remodeling of Genetic Molecules

The maternal HPA axis undergoes dramatic changes following conception, with cortisol levels rising to three times above the non-pregnant level by late pregnancy. Under normal conditions, the fetal-placental glucocorticoid inactivating enzyme 11β-hydroxysteroid dehydrogenase type 2 (HSD11B2) protects the fetus from excessive maternal cortisol levels (Duthie & Reynolds, 2013). However, maternal HSD11B2 activity may be altered by maternal stress and disease. This alteration may contribute to increased glucocorticoid transfer from mother to fetus. Elevated glucocorticoid exposure during fetal development is associated with adverse birth outcomes, including lower birth weight and reduced gestational duration. This risk becomes further apparent in adulthood, which may result in altered HPA axis activity and an increased risk of mental illness. This phenomenon, termed “programming,” is thought to permanently alter physiological functions (Seckl & Holmes, 2007; Solano et al., 2016). Epigenetic mechanisms refer to heritable changes that do not alter DNA sequences but are mediated by DNA methylation, histone modifications, and microRNA (miRNA) pathways (L. Zhang et al., 2020). Current evidence for epigenetic mechanisms comes from human observational studies and experimental animal research. Although human studies have linked trauma exposure to altered DNA methylation patterns, direct evidence for stable transgenerational epigenetic inheritance remains limited.

DNA methylation is an important epigenetic mechanism in the intergenerational transmission of childhood abuse. Epigenetic changes induced by childhood abuse primarily mediate alterations in acquired behavioral traits in humans via targeting genes involved in the HPA axis (such as NR3C1 and FKBP5) (Stenz et al., 2018). For example, researchers examined postmortem brains of suicide victims and observed specific methylation patterns of the glucocorticoid receptor among those with a history of childhood abuse. In contrast, this pattern was not observed in suicide victims without childhood abuse histories. This methylation correlates with reduced glucocorticoid receptor expression, which may increase stress responses (McGowan et al., 2009). Studies have shown that offspring of Holocaust survivors exhibit significantly lower methylation levels of FKBP5 introns 6 and 7 compared with control groups. Lower methylation at these sites is associated with reduced self-reported anxiety symptoms, suggesting a potential protective mechanism (Bierer et al., 2020). Recent studies have also found that maternal ACEs are associated with reduced DNA methylation near the SCG5 gene promoter in their offspring—a gene associated with neuroendocrine function (Folger et al., 2022). Paternal childhood trauma and social instability have been linked to elevated OXTR methylation, which in turn reduces father involvement (Brown et al., 2020), suggesting an epigenetic pathway for paternal intergenerational transmission.

Beyond epigenetic mechanisms, the biological effects of a parental traumatic experience on the next generation may also be associated with the presence of specific alleles. For example, studies indicate that mothers with a history of childhood adversity who carry the G allele of OXTR rs53576—and who suffer from mood disorders—tend to process their infants’ negative emotional stimuli differently, a pattern that could be associated with offspring development in some cases. (Hiraoka & Nomura, 2019). Furthermore, while maternal history of childhood adversity may be associated with her offspring’s emotions, this association may be moderated by the offspring’s 5-HTTLPR genotype (Bouvette-Turcot et al., 2015). These findings offer important genetic and molecular biological perspectives for understanding transgenerational effects of traumatic experiences. Therefore, epigenetic findings should be interpreted as biologically plausible mechanisms rather than definitive proof of causal intergenerational inheritance.

## 4. Intervention

To break the impact of childhood trauma on neuropsychological disorders and its intergenerational transmission, we propose a dual-level, multi-perspective intervention framework based on the EPPM model. At the trauma level, interventions are further divided into individual-focused and relationship-focused approaches. For individual-focused intervention, group mindfulness-based cognitive therapy (MBCT) and cognitive behavioral therapy (CBT) effectively improve emotion regulation and social functioning in adolescents with mood or psychosis symptoms, particularly those with high adversity (Weintraub et al., 2023). In addition, adding structured components to home visitation effectively reduces maternal stress and PTSD symptoms (van der Stouwe et al., 2023). For relationship-focused intervention, Parent–Child Interaction Therapy (PCIT) reduces parenting stress, negative parenting, child behavioral problems, and trauma symptoms, while lowering abuse recidivism—directly interrupting the transmission of maladaptive parenting (Warren et al., 2022). Furthermore, family-centered substance use treatment effectively improves family relationships and reduces adverse childhood experiences in offspring (Morgan et al., 2024).

At the neuropsychiatric level, interventions include physical interventions (e.g., transcranial magnetic stimulation (TMS), transcranial direct current stimulation (tDCS)) and pharmacological interventions. For physical interventions, TMS is effective for adolescent depression, with an overall response rate of 41.3% (Sigrist et al., 2022). TMS and tDCS also show significant efficacy across multiple adult mental disorders, including depression and PTSD (Sabé et al., 2024). Electroconvulsive therapy (ECT) remains effective for many, but poorer response in those with childhood sexual abuse highlights the importance of tailoring neurostimulation to trauma history (Thompson et al., 2024). For pharmacological interventions, fluoxetine significantly reduces pro-inflammatory cytokines in depressed adolescents with childhood trauma, though residual symptoms suggest a need for adjunctive anti-inflammatory strategies (Y. Li et al., 2026). Together, these trauma-level and neuropsychiatric interventions provide a dual pathway to disrupt intergenerational transmission.

Together, these trauma-level and neuropsychiatric interventions provide a dual pathway to disrupt intergenerational transmission.

## 5. Summary and Outlook

The effects and mechanisms of intergenerational transmission of childhood trauma have attracted significant attention. This paper systematically reviews the far-reaching consequences of childhood trauma and its specific intergenerational transmission mechanisms. Drawing on a multidisciplinary perspective encompassing negative parenting patterns, neurophysiological dysfunction, the intrauterine environment, inflammatory mechanisms, and genetic and molecular pathways, we aim to propose intervention strategies that provide a solid theoretical foundation for promptly breaking this vicious cycle of trauma.

Several limitations should be acknowledged. First, this review focuses primarily on transmission mechanisms and does not systematically examine protective factors, resilience processes, intervention strategies, or cultural influences that may buffer intergenerational risk. Second, although the EPPM model conceptualizes transmission as a dynamic developmental process, current evidence remains limited regarding bidirectional interactions among mechanisms and the extent to which pathways mutually influence one another across development. Third, important parental and offspring sex differences remain insufficiently understood. Existing studies have focused predominantly on maternal childhood trauma, while paternal transmission pathways, as well as potential father–son, father–daughter, mother–son, and mother–daughter differences, have received considerably less attention. Finally, the strength of evidence varies across mechanisms. While inflammatory and epigenetic pathways are increasingly supported by experimental animal studies that permit stronger causal inference, evidence for behavioral, neurophysiological, and intrauterine mechanisms remains largely observational and therefore requires further causal validation.

The EPPM model also highlights several priorities for future research. First, future studies should clarify how environmental, psychological, physiological, and molecular mechanisms interact over time, including potential feedback loops, reciprocal influences, and cross-level developmental processes. Second, greater attention should be devoted to parental and offspring sex differences, particularly the unique contributions of paternal childhood trauma and the possibility that transmission processes differ according to parent–child gender combinations. Third, interdisciplinary research combining prospective longitudinal designs, neuroimaging, biological assessments, experimental approaches, and animal models is needed to strengthen causal inference for behavioral, neurophysiological, and intrauterine pathways. Finally, future work should move beyond risk-focused models to incorporate resilience, protective factors, and intervention targets, thereby facilitating the development of evidence-based strategies to interrupt the intergenerational transmission of childhood trauma. Multi-generational cohort studies and advanced developmental modeling approaches may further contribute to refining and empirically validating the EPPM framework.

## Figures and Tables

**Table 1:** Keywords and themes.

Mechanism	Keywords (Combined with Boolean Operators)
1. Transmission of negative parenting behaviors	((parenting style) OR (negative parenting) OR (child maltreatment) OR (parent–child interaction) OR (emotional abuse) OR (physical abuse) OR (neglect)) AND ((intergenerational transmission) OR (childhood trauma) OR (Adverse childhood experiences))
2. Neural encoding of traumatic stress	((brain structure) OR (amygdala) OR (hippocampus) OR (prefrontal cortex)) OR (HPA axis) OR (cortisol) OR (stress reactivity) OR (CRH) OR (ACTH)) AND ((childhood trauma) OR (early life stress) OR (Adverse childhood experiences))
3. Intergenerational neurophysiological foundations	((mother-infant similarity) OR (parent–child neural similarity) OR (intergenerational transmission)) AND ((functional connectivity) OR (cortical thickness))
4. Biological permeation of the intrauterine environment	((maternal immune activation) OR (MIA) OR (pro-inflammatory cytokines) OR (IL-6) OR (placenta) OR (fetal brain development)) AND (prenatal stress)
5. Epigenetic remodeling	((DNA methylation) OR (histone modification) OR (miRNA) OR (NR3C1) OR (FKBP5) OR (11β-HSD2)) AND ((childhood trauma) OR (intergenerational) OR (Adverse childhood experiences))

## Data Availability

No new data were created or analyzed in this study. Data sharing is not applicable to this article.

## Abbreviations

The following abbreviations are used in this manuscript:

CT	childhood trauma
EA	emotional abuse
PA	physical abuse
EN	emotional neglect
PN	physical neglect
ADHD	attention deficit hyperactivity disorder
PTSD	post-traumatic stress disorder
ACEs	adverse childhood experiences
HPA	hypothalamic–pituitary–adrenal
